# Syrah Grape Polyphenol Extracts Protect Human Skeletal Muscle Cells from Oxidative and Metabolic Stress Induced by Excess of Palmitic Acid: Effect of Skin/Seed Ripening Stage

**DOI:** 10.3390/antiox13030373

**Published:** 2024-03-19

**Authors:** Adriana Capozzi, Auriane Dudoit, Luca Garcia, Gilles Carnac, Gérald Hugon, Cédric Saucier, Catherine Bisbal, Karen Lambert

**Affiliations:** 1 PhyMedExp, INSERM U1046, CNRS UMR 9214, University of Montpellier, CEDEX 5, 34295 Montpellier, France; adriana.capozzi@umontpellier.fr (A.C.); gilles.carnac@inserm.fr (G.C.); gerald.hugon@inserm.fr (G.H.); 2 SPO, INRAE, Institute Agro, University of Montpellier, 34000 Montpellier, France; auriane.dudoit@umontpellier.fr (A.D.); luca.garcia@umontpellier.fr (L.G.); cedric.saucier@umontpellier.fr (C.S.)

**Keywords:** antioxidants, grape polyphenols, ripening stage, grape tissue, mitochondrial activity, insulin resistance, human skeletal muscle

## Abstract

Metabolic skeletal muscle (SM) dysfunction, triggered by increased oxidative stress and mitochondrial impairment, is a pivotal contributor to obesity-associated insulin resistance (IR). Addressing obesity and SM IR demands substantial lifestyle changes including regular exercise and dietary adjustments that are difficult to follow over time. This prompted exploration of alternative approaches. Grape polyphenols (GPPs) have demonstrated a positive impact on metabolism, although few studies have focused on SM. Since grape polyphenolic content and composition depend on tissue and ripening, we explored the antioxidant potential of GPPs from skin (Sk) and seeds (Sd) extracted before veraison (Bv) and at mature (M) stages, on palmitate-induced IR in primary human SM cells. Despite their important difference in polyphenol (PP) content: Sd-BvPP > Sd-MPP/Sk-BvPP > Sk-MPP, all extracts reduced lipid peroxidation by 44–60%, up-regulated the heme-oxygenase 1 protein level by 75–132% and mitochondrial activity by 47–68%. Contrary to the other extracts, which improved insulin response by 50%, Sd-BvPP did not. Our findings suggest that compounds other than stilbenoids or anthocyanin-type molecules, present only in grape Sk, could play an active role in regulating SM oxidative and metabolic stress and insulin sensitivity, paving the way for further exploration of novel bioactive compounds.

## 1. Introduction

The current obesity pandemic is a significant public health issue, affecting one-third of the global adult population. Obesity, body mass index (BMI) ≥ 30 kg/m^2^, is associated with an increased risk of several diseases such as insulin resistance (IR), the central feature of type 2 diabetes (T2D). IR results in deficient glucose homeostasis and altered mitochondrial oxidative capacity. SM is a major regulator of glucose homeostasis and SM dysfunction is a primary component of obesity-induced IR [1,2]. During obesity, nutritional excess due to dietary fat intake is associated with dyslipidemia and ectopic fat accumulation in SM. SM IR strongly correlates with intramyocellular lipid content [3]. The increased availability of intramyocellular lipids represents an excess of fuel stimulating SM mitochondrial activity. Consequently, oxidative phosphorylation increases, inducing higher reactive oxygen species (ROS) production and emission [3]. The increase in mitochondrial ROS is one of the early events leading to SM dysfunction and to IR and T2D [4]. Under physiological conditions, ROS are essential intracellular signalers and the dynamic balance between ROS production and antioxidant defense systems maintains cellular redox homeostasis. Thus, mitochondria are critical organelles and preserving both their quantity and activity is essential for maintaining muscle metabolic homeostasis [5] and muscle health [6]. During obesity, lipid overload overwhelms mitochondrial activity [3,7], leading to lipid accumulation and higher ROS production that surpass the capacity of antioxidant defenses for their neutralization. This imbalance favors alteration in redox homeostasis and muscle metabolism, harming cellular components and impairing their functions [8,9]. ROS-induced modifications include peroxidation of polyunsaturated membrane lipids and formation of 4-hydroxy-2-nonenal aldehyde (4-HNE), which binds DNA, lipids and proteins, thereby inducing their dysfunction. Mitochondrial polyunsaturated membrane lipids are the primary target of ROS attack [10]. This leads to mitochondrial alteration, characterized by reduced activity of citrate synthase (CS)—a pivotal enzyme in the citric acid cycle—and cytochrome c oxidase subunit IV (CoxIV), an integral part of the mitochondrial electron transport chain [3,7]. These alterations associated with lipid overload result in disruption of signaling cascades and IR development [11].

The nuclear factor erythroid 2-related factor 2 (Nrf2) molecular pathway is crucial for suppressing oxidative stress and restoring homeostasis. The transcriptional factor Nrf2 is the final effector of a cellular pathway activated by the phosphorylation of the ubiquitin-binding protein p62 at serine 349 (P-p62). In fact, P-p62 interacts with the Nrf2 inhibitor, the Kelch-like ECH-associated protein 1 (Keap1), culminating in its degradation by the proteasome and provoking translocation of Nrf2 to the nucleus and transcription of its target genes [12]. Nrf2 promotes the expression of antioxidant enzymes such as heme-oxygenase 1 (HO-1), catalase and superoxide dismutases (SODs) [13]. Importantly, it has recently been shown that HO-1 could regulate mitochondrial functions and biogenesis during exercise and nutritional stress and drive insulin sensitivity [14,15]. Thus, the Nrf2 pathway has emerged as a master regulator of redox metabolism and a central mechanism for controlling and protecting mitochondrial metabolism [16].

Tackling obesity and SM IR requires significant lifestyle modifications like exercise and dietary changes. However, maintaining these behavioral modifications for the long-term can be challenging, inducing consideration of medications and surgery despite their associated side effects and post-surgery risks. The pressing need for new strategies to prevent and treat obesity and IR has prompted exploration of alternative approaches. Recent research studies have highlighted the potential antioxidant properties of phytonutrients found in plants, vegetables and fruits such as grape [17,18,19]. Grape, undoubtedly one of the most widely consumed fruits globally, is a noteworthy source of polyphenols, offering an impressive array of both quantity and variety. Grape polyphenols (GPPs) possess diverse biological effects and have demonstrated positive impacts on obesity, oxidative stress, mitochondrial activity and insulin sensitivity in both in vitro and in vivo settings [20,21,22]. The Syrah grape, also known as Shiraz in some regions, holds significant importance in the world of wine thanks to its versatility and ability to deliver high quality wines. This is why it is highly cultivated all over the world, although more extensively in the Mediterranean area. Wine production results in the generation of significant quantities of wine by-products. Wine by-products are essentially composed of grape pomace, a mixture of stems, Sk and Sd [18]. Interestingly, grape Sk and Sd are the grape’s tissues where the polyphenols are mainly concentrated [23,24,25]. However, it must be noted that the GPPs content is dependent on both tissue and ripening [26]. Flavanols (or flavan-3-ols) are mainly contained in grape Sd and in Sk in lower concentrations. Their concentration increases with ripening while their degree of polymerization (mDP), i.e., the proanthocyanidins’ extension, reaches its peak a few weeks Bv. Anthocyanins are only found in grape Sk, to which they give the characteristic color. Their concentrations undergo a marked increase during the later stages of ripening. Flavonols, another important subgroup of flavonoids, are highly concentrated in grape Sk, albeit in lower concentrations than anthocyanins, with concentrations peaking during the mid to late stages of ripening. Lastly, stilbenes, the most renowned group of GPPs for their diverse biological activities, are accumulated during the later stages of ripening essentially in grape Sk, and in lower concentrations in Sd.

However, studies of GPP activities on SM insulin sensitivity are still sparse [27] and to our knowledge, despite the fact that grapes contain high concentrations of PPs at the Bv stage [28], there are no studies exploring their impact on muscle metabolism at this stage. Our previous results showed that Bv GPPs possess a high anti-inflammatory potential [28] and inhibit the α-glucosidase enzyme in vitro [29], therefore being an excellent candidate for T2D regulation.

Accordingly, our investigation aimed to examine the antioxidant and metabolic effects of polyphenols extracted from grape Sk and Sd at Bv and M stages in a well-established model of IR primary human SM cells [7]. Our objective was to identify the more efficient extract against SM alteration in obesity.

## 2. Materials and Methods

### 2.1. Grape Materials

Samples of Syrah grape were collected from the Institut National de Recherche pour l’Agriculture, l’Alimentation et l’Environnement (INRAE) vineyard of Montpellier at two different ripening stages: Bv (18 June 2018) and M (3 September 2018) (43°37′02.7″ N, 3°51′22.3″ E, annual mean temperature: 16.38 °C, average annual rainfall: 1063.5 mm and soil-type gravel and river sand). After harvest, grape bunches were quickly frozen at −80 °C pending extracts preparation. 

### 2.2. Determination of Total Phenolic Content and Total Antioxidant Capacity

Total phenolic content (TPC) was measured using the Folin–Ciocalteu colorimetric method and expressed as mg of gallic acid equivalent (GAE)/g of dry extract. Total antioxidant capacity was determined by 2,2-diphenyl-1-picrylhydrazyl (DPPH) radical scavenging assay and expressed in µmol of Trolox equivalent (TE)/g of dry extract.

### 2.3. Qualitative Profiling of GPP Extracts

#### 2.3.1. Anthocyanins

Ultra-performance liquid chromatography (UPLC) anthocyanin analysis was carried out as previously described and expressed as malvidin-3-*O*-glucoside equivalent/L [28].

#### 2.3.2. Stilbenes

Stilbenes analysis was carried out following a previous report [28]. GPP samples (20 g/L in 50:50 *v*/*v* methanol/water) were analyzed with UPLC with the mobile phases consisting of solvent A (water/ trifluoroacetic acid 99:1 *v*/*v*) and solvent B (acetonitrile 100%) using the following gradients: 0 min 5% B, 1 min 5% B, 15 min 20% B, 16 min 95% B, 18 min 95% B and 20 min 5% B. Calibration curves of trans-resveratrol and trans-piceid were used for quantification and results are expressed as mg of their respective standards.

#### 2.3.3. Monomeric Flavan-3-ols and Proanthocyanidins

UPLC analysis of proanthocyanidins was carried out following a previously described protocol [28] and expressed as mg equivalent of catechin hydrate/g of dry extract. In order to calculate the mDP, the sum of all subunits (flavan-3-ol monomers and phloroglucinol adducts expressed in millimoles) was divided by the sum of all flavan-3-ols monomers (expressed in millimoles).

### 2.4. Human Primary SM Cell Culture and Treatment

Human primary SM cells were isolated from left vastus lateralis biopsies as previously described [30]. Human SM cells were plated at 10^4^ cells/cm^2^ cell density and after 48 h were treated or not with 750 μM palmitate [31] (Sigma-Aldrich, ST Quentin Fallavier, France) in the presence or not of GPP extracts at the indicated concentration, for 24 h. For the insulin response, human SM cells were incubated with or without human insulin 0.6 μM (Umuline RAPIDE; Lilly, Neuilly-sur-Seine, France) during 10 min at 37 °C before harvesting.

### 2.5. Western Blot Analysis

After treatment, human SM cells were lysed in an SDS-PAGE sample buffer, and proteins were analyzed by Western blotting. Membranes were incubated overnight with antibodies against pan-actin, HO-1, p62, Pser349-p62 (P-p62), CS, CoxIV, sod1, catalase, Akt, Pser473-Akt (P-Akt) (all from Cell Signaling Technology, Ozyme, Saint-Quentin-en-Yvelines, France), glutathione reductase (GR) (AbFrontier, VWR, Rosny-sous-Bois, France), 4-HNE (Abcam, Paris, France) and then with species-directed secondary antibodies conjugated to IRDye800 or IRDye680 (LI-COR Biosciences, Bad Homburg, Germany). Membranes were analyzed with the Odyssey CLx Imaging System (LI-COR Biosciences, Bad Homburg, Germany). Protein expression was quantified using ImageJ software (Version 1.42q/Java1.6.0_14, National Institutes of Health, New York, NY, USA, http://rsbweb.nih.gov/ij/index.html, accessed on 31 January 2024). Protein quantification of each sample was corrected by the quantification of the corresponding pan-actin, analyzed on the same sample and same membrane. Data are expressed relative to the human SM cells mean level of protein expression in palmitate conditions without GPP extract treatment, which was set at 1.

### 2.6. GPP Extracts Toxicity/Cell Viability

Human SM cells viability after GPP extract treatment was determined with PrestoBlue^®^ assay (Invitrogen, ThermoFisher Scientific, Illkirch Cedex, France), following the manufacturer’s instructions. Data were expressed relative to the mean level in the control non-treated human SM cells, which was set at 100%.

### 2.7. Citrate Synthase Activity

CS activity was monitored in 1 µL of total human SM cell extract as previously described [20]. CS activity was normalized to total protein amount and expressed as µmol/min/µg of protein.

### 2.8. Statistical Analysis

Multiple group comparisons were performed using one-way ANOVA followed by Tukey’s post-hoc test. Two groups’ comparisons were performed using Student’s *t*-test. All analyses were performed with GraphPad PRISM 6 software. For all tests, we considered that significance was reached when *p* ≤ 0.05 with * *p* ≤ 0.05, ** *p* < 0.01, *** *p* < 0.001 and **** *p* < 0.0001. Values were expressed as mean ± standard error of the mean (SEM) of three independent experiments. 

## 3. Results

### 3.1. Characterization of Human SM Cells’ Metabolic Alterations Induced by Lipid Overload

**Figure 1:**
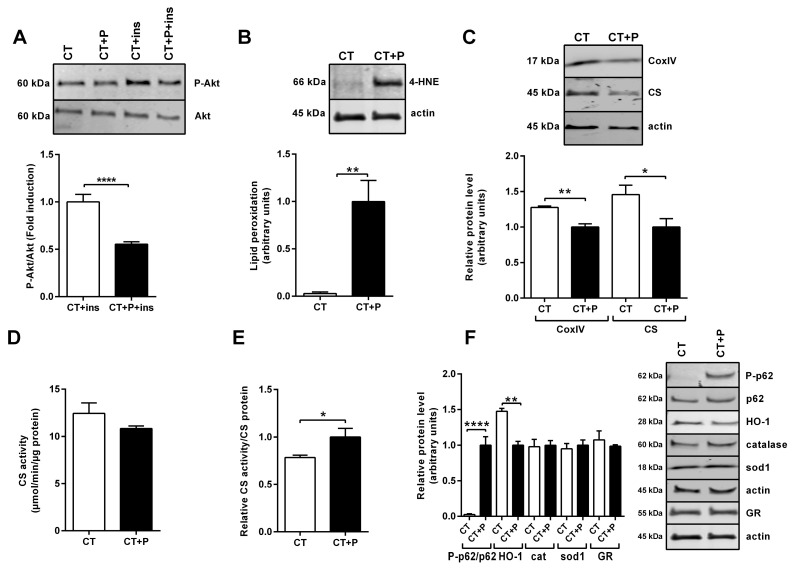
Metabolic dysfunction of human SM cells following palmitate treatment. (**A**) Representative Western blot of P-Akt and Akt following insulin (ins) stimulation of human SM cells treated or not with palmitate (P). P-Akt/Akt level expressed as fold induction relative to baseline level of P-Akt/Akt without palmitate treatment that was set at one. (**B**) Representative Western blot analysis of human SM cell protein 4-HNE adducts following palmitate treatment. The 4-HNE-protein levels are expressed relative to actin and to 4-HNE-protein levels following palmitate treatment that was set at one. (**C**) Representative Western blot of CoxIV and CS protein expression analysis in human SM cells following palmitate treatment. Protein levels were expressed relative to actin level and to their level in palmitate-treated cells that was set at one. (**D**) CS enzymatic activity in human SM cells treated or not with palmitate. (**E**) CS activity relative to CS protein. (**F**) Representative Western blot analysis of P-p62, p62, HO-1, catalase, sod1 and GR in human SM cells treated or not with palmitate. P-p62/p62 levels are expressed as fold induction relative to P-p62/p62 level induced by palmitate treatment that was set at one. Levels of other proteins were expressed relative to actin level and to their level in palmitate-treated cells that was set at one. For all the experiments, n = 3. Data are expressed as means ± SEM. Statistical significance was determined by Student’s *t*-test. * *p* ≤ 0.05, ** *p* < 0.01 and **** *p* < 0.0001.

Palmitic acid (palmitate) is one of the most abundant saturated fatty acids within the body and present in food. As such, it can be supplied by the diet or produced endogenously via de novo lipogenesis [32]. Consequently, the use of palmitate-treated cells has been extensively used to study IR and has been shown to be an appropriate model [7]. To mimic overfeeding and lipid overload occurring in obesity, human SM cells were treated with palmitate for 24 h [7,8,33]. Insulin response, measured as the fold induction of the ratio of phosphorylated ser493Akt (P-Akt) to non-phosphorylated Akt (P-Akt/Akt) with and without insulin and palmitate, was significantly reduced in palmitate-treated cells compared to control non-treated cells (Figure 1A). Induction of oxidative stress was evidenced by protein adducts of 4-HNE, which increased after palmitate treatment (Figure 1B). However, we did not observe any protein carbonylation under these conditions. While CS and CoxIV protein levels (Figure 1C) were significantly reduced after palmitate treatment, there was no modification in CS activity (Figure 1D). Consequently, the ratio between CS activity and CS protein amount was significantly increased after 24 h of palmitate treatment (Figure 1E). A high induction of phosphorylated ser349p62 (P-p62) to non-phosphorylated p62 (P-p62/p62) ratio was observed, though without subsequent pathway activation, as evidenced by the lowered expression of the downstream antioxidant effector HO-1 and no induction of catalase or sod1 proteins (Figure 1F). In the same way, the amount of an essential cellular redox regulator, the enzyme glutathione reductase (GR), was not altered after palmitate treatment (Figure 1F).

### 3.2. Grape Extracts Characterization and Cell Viability

**Figure 2:**
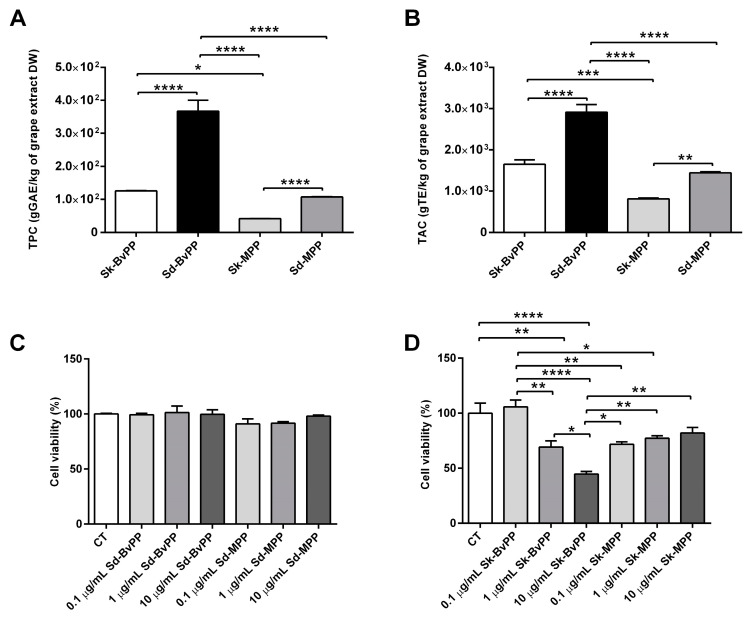
GPP extract antioxidant activity and cellular toxicity. (**A**) TPC of GPP extracts measured by the Folin–Ciocalteu method. (**B**) TAC of GPP extracts measured by the DPPH assay. (**C**) Sd-GPP extract toxicity on human SM cells at increasing concentrations (0.1–10 µg/mL). (**D**) Sk-GPP extract toxicity on human SM cells at increasing concentrations (0.1–10 µg/mL). For all the experiments, n = 3. Data are expressed as means ± SEM. Statistical significance was determined via one-way ANOVA. * *p* ≤ 0.05, ** *p* < 0.01, *** *p* < 0.001 and **** *p* < 0.0001.

First, the composition and potential cell toxicity of GPP extracts were determined. These extracts presented different qualitative and quantitative PP composition (Table 1 and Table 2) in relation to grape tissues and ripening stage. The quantification of anthocyanins in grape Sd was not pursued, as these compounds are not known to be present in this particular tissue [28]. Analysis of monomeric flavan-3-ols was accompanied by a measure of the mDP of the proanthocyanidins. Sd extracts contained higher amounts of flavan-3-ols than Sk extracts, both decreasing with ripening. Flavan-3-ols were less polymerized in grape Sd compared to Sk. Additionally, the analysis indicated that the mDP of flavan-3-ols tended to decrease during the ripening process, leading to a reduction in proanthocyanidins in both Sd and Sk tissues (Table 1). Sk-MPP extracts wee rich in anthocyanins (Table 2). Stilbenes were also quantified in Sk extracts and their concentration increased as the grape ripened (Table 2). The highest total phenolic content (TPC) was observed at the Bv stage both in Sk and Sd extracts (Figure 2A) and significantly decreased to reach a minimum value at M stage (Figure 2A). Total antioxidant capacity (TAC) showed a similar trend; the highest value was found at Bv stage for both Sk and Sd extracts with a significant reduction at M stage (Figure 2B).

Human SM cells were treated with increasing concentrations (0.1–1–10 µg/mL) of the GPP extracts over 24 h. Neither Sd-BvPP nor Sd-MPP extracts (Figure 2C) affected cell viability of human SM cells regardless of the concentrations tested. Sk-BvPP extract produced a 27% and 52% decrease in cell viability at doses of 1 and 10 µg/mL, respectively. Sk-MPP generated a 28% decrease in viability at all concentrations tested. We opted to operate at the concentration exhibiting the lowest toxicity for all the extracts: 0.1 µg/mL.

### 3.3. Effect of GPP Extracts on Antioxidant Response in Lipid-Overload Human SM Cells

**Figure 3:**
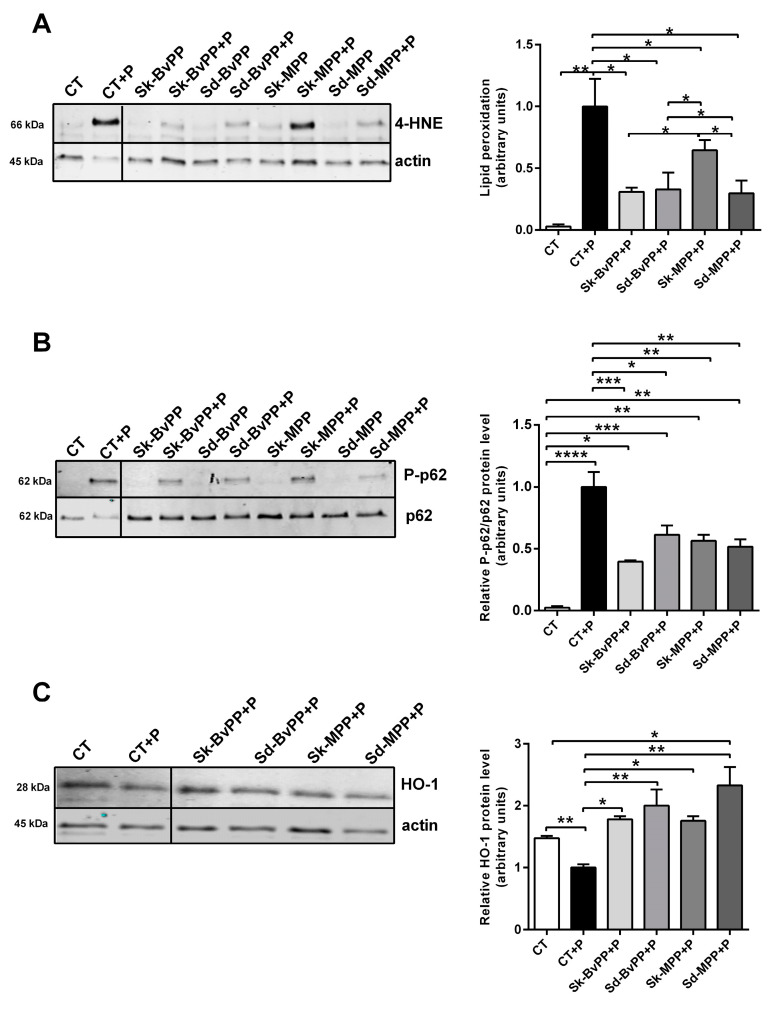
Treatment with GPP extract improves antioxidant response and decreases oxidative stress of human SM cells treated with palmitate. (**A**) Representative Western blot analysis of lipid peroxidation alteration after palmitate and GPP extract treatment of human SM cells. Protein 4-HNE adducts quantification is expressed relative to actin level and to their level in human SM cells treated with palmitate alone that was set at one. (**B**) Representative Western blot analysis of P-p62 and p62 proteins in human SM treated or not with palmitate and GPP extracts. P-p62/p62 levels are expressed as fold induction relative to P-p62/p62 levels induced by palmitate treatment that was set at one. (**C**) Representative Western blot analysis of HO-1 protein expression after GPP extract and palmitate treatment. Protein levels were expressed relative to actin level and to their levels in palmitate-treated human SM cells that was set at one. For all the experiments, n = 3. Data are expressed as means ± SEM. Statistical significance was determined by one-way ANOVA. * *p* ≤ 0.05, ** *p* < 0.01, *** *p* < 0.001 and **** *p* < 0.0001.

As shown in Figure 3A, treatment of human SM cells with GPP extracts produced a significant decrease in palmitate-induced lipid peroxidation if compared to GPP-untreated cells. When GPP extracts were added to palmitate, a lower phosphorylation of p62 compared to palmitate alone was observed (Figure 3B). While the intense phosphorylation in palmitate-treated human SM cells was accompanied by a decrease in HO-1 expression, the latter increased with GPP extract treatment compared to palmitate-treated cells (Figure 3C).

### 3.4. Effect of GPP Extracts on Human SM Cells Mitochondrial Activity and Insulin Response

**Figure 4:**
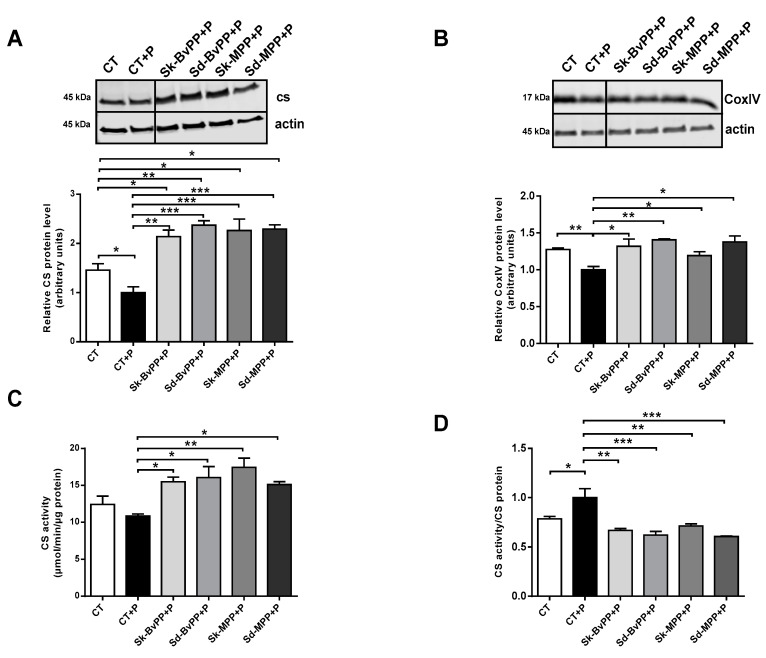
Treatment of human SM cells with GPP extract concomitantly to palmitate treatment improves their mitochondrial activity. Representative Western blot of CS (**A**) and CoxIV (**B**) protein expression analysis following treatment of human SM cells with palmitate and GPP extracts. Protein levels were expressed relative to actin level and to their levels in palmitate-treated human SM cells that was set at one. (**C**) CS activity of human SM cells treated with or without palmitate and GPP extracts. (**D**) Ratio between CS activity and CS protein level measured by Western blot. * *p* ≤ 0.05, ** *p* < 0.01, *** *p* < 0.001.

**Figure 5:**
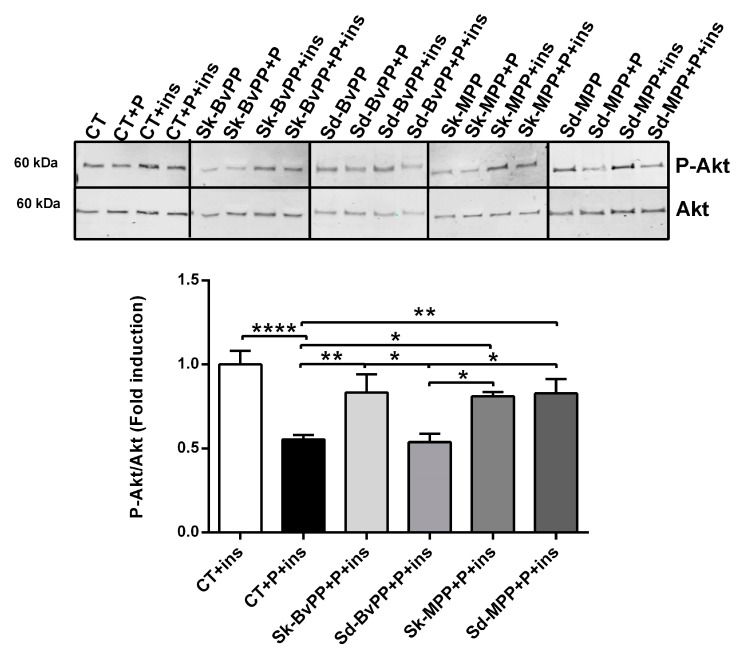
Treatment of human SM cells with GPP extracts concomitantly to palmitate treatment improves insulin response except for Sd-BvPP extract. Representative Western blot of P-Akt and Akt following insulin stimulation of human SM cells treated or not with palmitate and GPP extracts. P-Akt/Akt levels are expressed as fold induction relative to baseline level of P-Akt/Akt fold induction without palmitate treatment that was set at one. n = 3. Data are expressed as means ± SEM. Statistical significance was determined via one-way ANOVA. * *p* ≤ 0.05, ** *p* < 0.01 and **** *p* < 0.0001.

All GPP extracts were able to significantly increase CS and CoxIV protein expression (Figure 4A,B) that was decreased after palmitate treatment. GPP extracts also increased CS activity (Figure 4C). After GPP extract treatment, the ratio between CS activity and CS protein amount was decreased compared to palmitate alone and reached the control level (Figure 4D). Interestingly, a significant increase in the insulin signaling pathway, measured by the rise of the P-Akt/Akt ratio following insulin stimulation, was obtained by treatment with all GPP extracts, except Sd-BvPP (Figure 5).

## 4. Discussion

This study represents the first evidence of the biological activity of Sk-BvPP and Sd-BvPP extracts on the metabolism of human SM cells. All GPP extracts tested whatever the tissue and ripening stage had the ability to reduce lipid oxidative stress and to increase the oxidative capacity of palmitate-treated human SM cells. These data shed light on the impact of the tissue of origin and ripening stage of grapes against early muscle alterations favoring IR and the potential benefits of GPPs in muscle metabolism.

Interestingly, while the TPC and TAC were higher in Sd compared to Sk and in the Bv stage compared to the M stage, it emerged that all extracts had comparable effects in boosting SM antioxidant response and oxidative capacity. The only exception was represented by the Sd-BvPP extract that, despite decreasing lipid oxidative stress and improving the oxidative capacity of palmitate-treated human SM cells, did not improve muscle insulin sensitivity. However, this observation implied that the efficacy of these extracts on insulin response was probably attributable to a more complex mechanism at play. Besides the direct antioxidant scavenging properties of GPP, they may synergistically/antagonistically interact with endogenous molecules to exert their biological activities [34]. As mentioned above, oxidative stress plays a pivotal role in the pathophysiology of SM IR and obesity, triggered by an excess of glucose and free fatty acids in the bloodstream, leading to heightened mitochondrial requirements, ineffectiveness of antioxidant systems and disease progression [35]. Therefore, mitigating oxidative stress becomes a crucial objective. This study demonstrated the ability of GPP extracts, regardless of the tissue type or ripening stage, to decrease palmitate-induced lipid peroxidation in human SM cells. These ROS by-products attack is closely associated with insulin sensitivity. In the condition of IR, an increase in lipid peroxidation is observed at systemic level [35], but also at an SM level [36]. On the contrary, any improvement in insulin sensitivity is accompanied by a significant reduction in 4-HNE which is similarly observed following supplementation with antioxidants or implementation of exercise [37]. Since 4-HNE can form covalent adducts with insulin [38] leading to the functional alteration of the Akt pathway [39], lowering 4-HNE levels improves insulin sensitivity. Accordingly, in our study, a reduction in SM cells 4-HNE associated with an increase in insulin sensitivity has been also found, although not of the same amplitude for all extracts. Actually, Sd-BvPP did not improve SM cells’ insulin sensitivity, whereas a lower 4-HNE level was observed compared to palmitate-treated cells, suggesting a nonlinear relation between these two parameters. Overall, the reduction in 4-HNE levels, compared to control palmitate-treated cells for Sk-BvPP, Sk-MPP and Sd-MPP, was sufficient to improve insulin sensitivity, although reaching a slightly higher level than the control cells. Metabolization of 4-HNE is achieved through GSH and glutathione-S-transferases; hence, an increase in metabolization could protect from aldehyde toxicity and muscle IR [11].

Furthermore, data displayed the enhanced activation of the Nrf2 antioxidant pathway. Treatment with palmitate led to a high phosphorylation of p62 in human SM cells, although not followed, as expected, by induction of the antioxidant HO-1. We can speculate a disrupted p62 activation leading to a decreased expression of HO-1, as has already been observed in obesity [40]. Treatment with GPP extracts increased HO-1 expression, overcoming the blockage and triggering this crucial cellular defense mechanism, essential for maintaining muscle health. Conversely, changes in cytosolic sod1 and catalase proteins levels were not observed. These data highlight the complexity and specificity of the extracts’ effects on the antioxidant system during metabolic stress due to 24 h palmitate treatment on SM cells. The main effects exerted by GPP extracts were therefore on muscle lipid peroxidation and mitochondrial enzymes’ expression and function. HO-1 has recently been identified as a regulator of mitochondrial function and biogenesis [14,15]. After treatment with all GPP extracts, a notable enhancement in both mitochondrial activity and quantity was observed, as evidenced by the increased activity of citrate synthase (CS) and the expression levels of both CS and CoxIV proteins. Furthermore, treatment with GPP extracts facilitated the normalization of the ratio between CS activity and CS protein levels. In human palmitate-treated SM cells, a decrease in CS protein expression was observed, while CS activity remained unchanged, suggesting an increase in activity per mitochondrion, as indicated by the elevated ratio of CS activity to CS protein. This elevation might signify an overactivity of mitochondria that increased the production of ROS, which was subsequently mitigated by GPP treatment.

Remarkably, Sd-BvPP demonstrated a reduction in lipid peroxidation, an enhancement in mitochondrial function and a reduction in oxidative stress, although without a reversal of IR caused by lipid overload in human SM cells. These data question the classical relationship between oxidative stress and IR, at least in SM. While this outcome may seem perplexing, it aligns with previous findings that have indicated grape Sd extracts could potentially impair insulin signaling pathways [41]. We can hypothesize various mechanisms to explain this phenomenon, such as another biological activity not yet identified regulating insulin response or the potential presence in the extract of a specific inhibitor of insulin signaling, yet not inducing SM cell toxicity as analyzed and shown in Section 3.2 (Figure 2C).

Our observations suggest that GPP extracts not only at the M stage, as extensively demonstrated in previous decades, but also those from the Bv stage enhance mitochondrial activity and biogenesis, as evidenced in palmitate-treated SM cells [20,21]. The qualitative and quantitative analysis conducted by means of UPLC revealed that the primary subclass of polyphenols in Sd extracts are flavan-3-ols (in monomer or polymerized form). Given the absence of anthocyanins and stilbenes in grape Sd extracts, molecules often attributed to the health-promoting properties of grapes [42,43,44], we can conclude that other polyphenols or classes of compounds are certainly present as bioactive molecules and require further exploration to profile them. It is now well known that PPs could be metabolized before their absorption and/or excreted from the body, although this is highly dependent on their molecular structures and GPPs show considerable variability. Furthermore, Sk-BvPP showed some cell toxicity at a high concentration, which could limit their use in vivo. Therefore, future evaluations of GPP toxicity, metabolism and bioavailability within a living organism will be necessary to validate our findings and ascertain the therapeutic potential and toxicity of the extract in a more physiologically relevant context. However, it is noteworthy that we observed metabolic and antioxidant effects at a low dosage in vitro, suggesting plausible translatability to in vivo settings, given our prior findings demonstrating the beneficial effects on humans induced by treatment with a mixture of GPPs from the M stage at the dose of 2 g/kg/d [20].

Here, our observations revealed that BvPP and MPP have the capacity to enhance SM metabolism, mitochondrial quantity and activity, thereby improving muscle function, as evidenced following physical activity. These compounds could be likened to “exercise mimetic pills”, providing a potential alternative or supplement to physical activity. Indeed, maintaining an active lifestyle marked by regular physical activity is crucial and must be promoted for an overall state of good health. However, exercise adherence tends to be generally low, and various factors such as illness, injuries and aging can often limit or hinder mobility. This is where “exercise-mimetic pills” can assume a pivotal role in replicating in the SM some of the beneficial effects of exercise on human health: improving oxidative metabolism, mitochondrial biogenesis and activity, insulin sensitivity and the cellular stress response among several other in vivo mechanisms [45,46,47].

## 5. Conclusions

This study provided the first evidence of the biological activity of Sk-BvPP and Sd-BvPP extracts on human SM cells’ metabolism. Notably, these extracts, along with Sk-MPP and Sd-MPP extracts, exhibit significant antioxidant properties and boost mitochondrial activity, both crucial in addressing the early muscle alterations associated with IR and obesity. 

Moreover, the study sheds light on the complex mechanism behind the biological effects of GPP extracts, since Sd-BvPP extract, despite exerting the above cited effects, was unable to improve insulin response, unlike all the other GPP extracts tested. The observed variations in activity are likely attributable to differences in PP composition and further research is needed to unravel the specific bioactive molecules responsible for the observed effects, knowing that these go beyond the classical stilbenoids or anthocyanin-like compounds. This work shows potential for uncovering novel molecules for the development of new pharmaceuticals or nutraceuticals derived from grapes. These advancements have the potential to contribute to the management of obesity and related conditions during their early stages.

## Figures and Tables

**Table 1:** Quantification of individual and polymerized flavan-3-ols in Sd and Sk extracts of Syrah grapes obtained at two different ripening stages. Results are expressed as mg of their corresponding standard per g of extract dry weight. * *p* < 0.05 significant difference between M and Bv stage. ^†^ *p* < 0.05 significant difference between Sk and Sd.

	Tissue			
	Sd		Sk	
	Ripening Stage		Ripening Stage	
Polyphenol Subclass	Bv	M	Bv	M
Flavan-3-ols				
(+)-Catechin	25.2 ± 0.31	29.4 ± 0.21 *	9.43 ± 0.16 ^†^	1.57 ± 0.12 *^,†^
(−)-Epicatechin	0.70 ± 0.03	28.8 ± 0.11 *	nd	0.33 ± 0.01 *^,†^
Epicatechin gallate	37.2 ± 0.08	22.3 ± 0.03 *	0.25 ± 0.01 ^†^	1.40 ± 0.36 *^,†^
Epigallocatechin	13.9 ± 0.19	0.88 ± 0.77 *	nd	nd
mDP	11.28 ± 0.17	5.38 ± 0.12 *	18.9 ± 0.22	11.6 ± 0.83 *^,†^

**Table 2:** Quantification of anthocyanins and stilbenes in Sk extract of Syrah grapes obtained at two different ripening stages. Results are expressed as mg of their corresponding standard per g of extract dry weight. nd: not detected. * *p* < 0.05 significant difference between M and Bv stage.

	Sk	
	Ripening Stage	
Polyphenol Subclass	Bv	M
**Anthocyanins**		
Delphinidin 3-*O*-Glucoside	nd	0.69 ± 0.01
Cyanidin 3-*O*-Glucoside	nd	0.29 ± 0.01
Petunidin 3-*O*-Glucoside	nd	0.95 ± 0.03
Peonidin 3-*O*-Glucoside	nd	1.86 ± 0.01
Malvidin 3-*O*-Glucoside	nd	4.59 ± 0.04
Delphinidin 3-*O*-Acetyl Glucoside	nd	0.13 ± 0.01
Cyanidin 3-*O*-Acetyl Glucoside	nd	0.05 ± 0.01
Petunidin 3-*O*-Acetyl Glucoside	nd	0.22 ± 0.01
Peonidin 3-*O*-Acetyl Glucoside	nd	0.76 ± 0.01
Malvidin 3-*O*-Acetyl Glucoside	nd	2.27 ± 0.05
Delphinidin 3-*O*-Coumaroyl Glucoside	nd	0.02 ± 0.01
Cyanidin 3-*O*-Coumaroyl Glucoside	nd	0.23 ± 0.01
Petunidin 3-*O*-Coumaroyl Glucoside	nd	0.33 ± 0.01
Peonidin 3-*O*-Coumaroyl Glucoside	nd	1.04 ± 0.02
Malvidin 3-*O*-Coumaroyl Glucoside	nd	2.02 ± 0.02
**Stilbenes**		
Trans-resveratrol	0.03 ± 0.01	0.19 ± 0.01 *
Cis-resveratrol	0.01 ± 0.01	0.03 ± 0.01 *
Trans-piceid	0.15 ± 0.01	1.01 ± 0.01 *
Cis-piceid	0.02 ± 0.01	0.21 ± 0.01 *

## Data Availability

Data are contained within the article.
